# Supplementary material for: Newly identified SpoVAF/FigP complex: the role in Bacillus subtilis spore germination at moderate high pressure and influencing factors
Source: Appl Environ Microbiol. 2025 Jan 21;91(2):e02047-24. doi: 10.1128/aem.02047-24 (PMC11837508; doi:10.1128/aem.02047-24)
Supplement: Supplemental material — Figures S1 to S6; Tables S1 to S3. [file aem.02047-24-s0001.pdf]

Supplementary Figure Legends

Figure S1. Total DPA content of *B. subtilis* PY79 (wt), YZ03 (Δ*figP*), and YZ05 (Δ*figP, amyE::figP*) spores. Spores (OD=0.5) were boiled for 20 min to determine total DPA content by monitoring the relative fluorescence units (RFU) of Tb^{3+}-DPA and then calculating the content based DPA standard curve. Data are represented as mean ± SD. Shown are the representative results of three independent biological experiments, each with three replicates.

Figure S2. Effects of the absence of SpoVAF or FigP on spore germination at MHP (200 MPa). (A) The percentage DPA release at 0, 1, 3, 5, 7, 10-min MHP treatment, relative to the total DPA content in *B. subtilis* PY79 (wt), YZ03 (Δ*figP*), and KP09 (Δ*spoVAF*) spores. (B) The percentage K^+ release at 1-min MHP treatment, relative to the total K^+ content in spores described in (A). Data are represented as mean ± SD. Shown are the representative results of three independent biological experiments, each with three replicates. One-way ANOVA with Tukey's multiple comparisons test was performed to compare the significant differences. Asterisks denote the significance levels: *$p < 0.05$.

Figure S3. Total DPA content of TYZ12 (Δ5, *amyE::gerA from B. subtilis*), YZR34 (Δ5 Δ*figP*, *amyE::gerA from B. subtilis*), TYZ13 (Δ5, *amyE::gerA from B. cereus*), and YZR37 (Δ5 Δ*figP*, *amyE::gerA from B. cereus*) spores. Spores (OD=0.5) were boiled for 20 min to determine total DPA content by monitoring the relative fluorescence units (RFU) of Tb^{3+}-DPA and then calculating the content based DPA standard curve. Data are represented as mean ± SD. Shown are the representative results of three independent biological experiments, each with three replicates.

Figure S4. Total DPA content of BLA201 (Δ5) and YZR33 (Δ5 Δ*figP*) spores.

Spores (OD=0.5) were boiled for 20 min to determine total DPA content by monitoring the relative fluorescence units (RFU) of Tb^{3+}-DPA and then calculating the content based DPA standard curve. Data are represented as mean ± SD. Shown are the representative results of three independent biological experiments, each with three replicates.

Figure S5. Effects of the absence of SpoVAF or FigP on spore germination at 200 and 500 MPa. Spores of BLA201 (Δ5), YZR33 (Δ5 Δ*figP*), YZR39 (Δ5 Δ*spoVAF*), KP33 (Δ5 Δ*figP figP*) and KP39 (Δ5 Δ*spoVAF spoVAF*) strains were treated with 200 and 500 MPa at 26°C to induce germination. DPA release was quantified by monitoring the relative fluorescence units (RFU) of Tb^{3+}-DPA, and the percentage of DPA release was calculated as the ratio of the released DPA content to total DPA content. Data are represented as mean ± SD. Shown are the representative results of three independent biological experiments, each with three replicates.

Figure S6. The protein-protein interaction network of SpoVAF and other SpoVA subunits predicted by STRING database. The likelihood of SpoVAF interacting with other subunits was quantified by combined-score, a thicker line indicated a higher prediction score and greater probability of interaction. The SpoVAF subunits (C, D, and Eb) that comprise the DPA release channel were highlighted in blue. SpoVAF exhibited a higher likelihood of interacting with these DPA channel forming subunits.

Figure S1

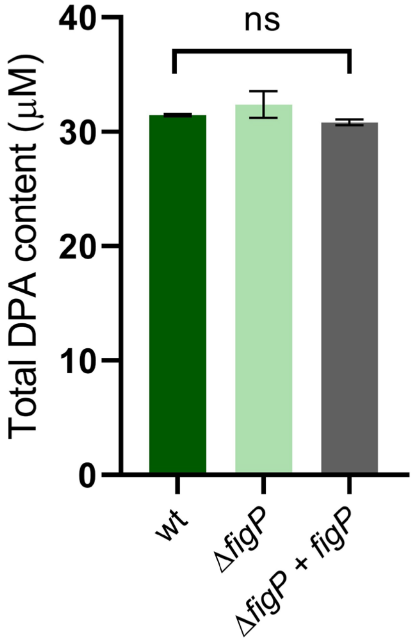

Figure S2

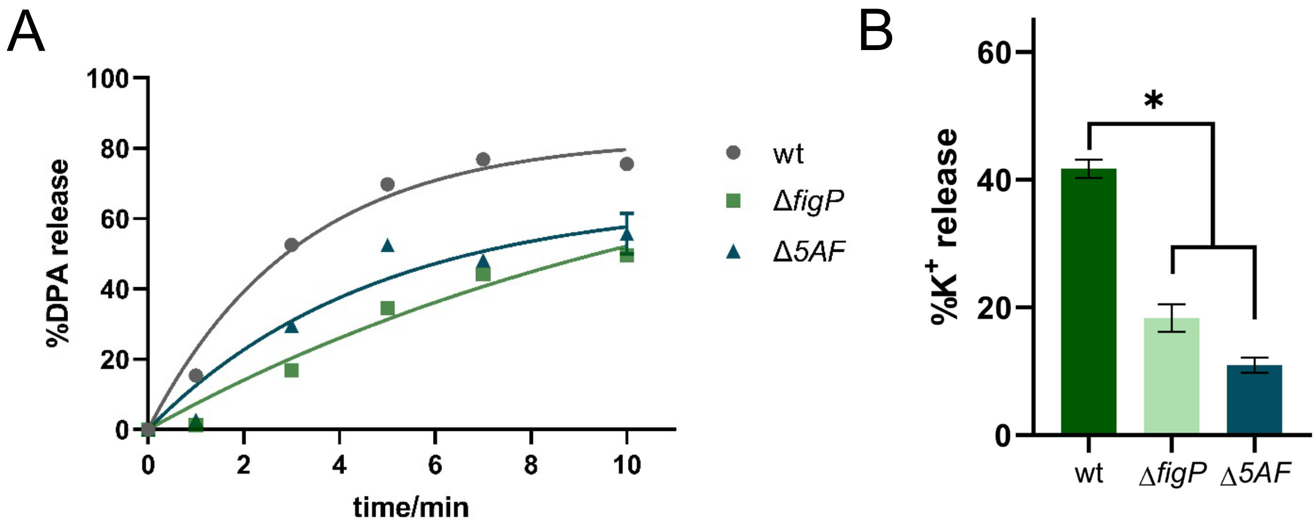
A
B
100
80
60
40
20
0
%DPA release
0
2
4
6
8
10
time/min
wt
ΔfigP
Δ5AF
60
40
20
0
%K+ release
*
wt
ΔfigP
Δ5AF

Figure S3

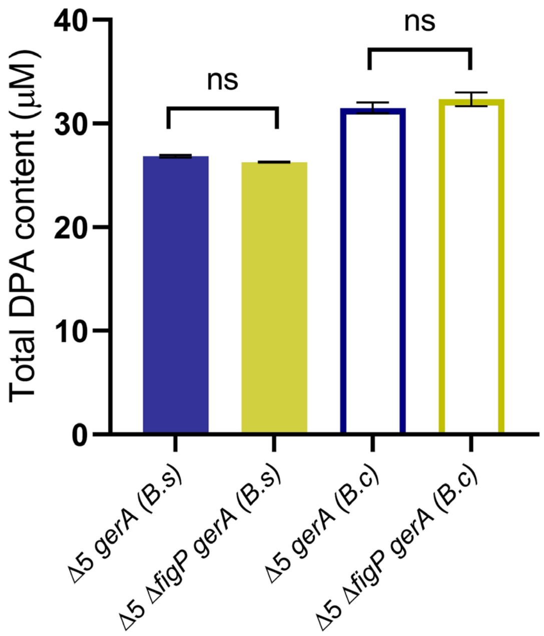

ns
ns
Total DPA content (µM)
40
30
20
10
0
Δ5 gerA (B.s)
Δ5 ΔfigP gerA (B.s)
Δ5 gerA (B.c)
Δ5 ΔfigP gerA (B.c)

Figure S4

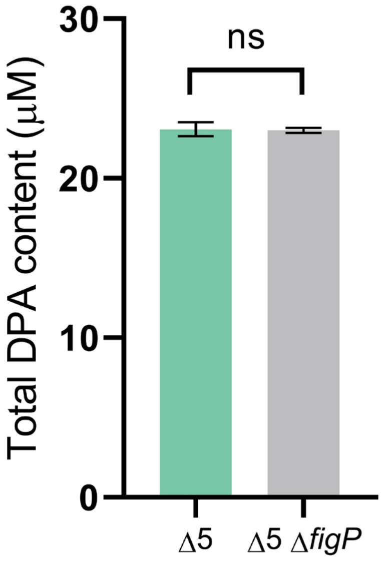

Figure S5

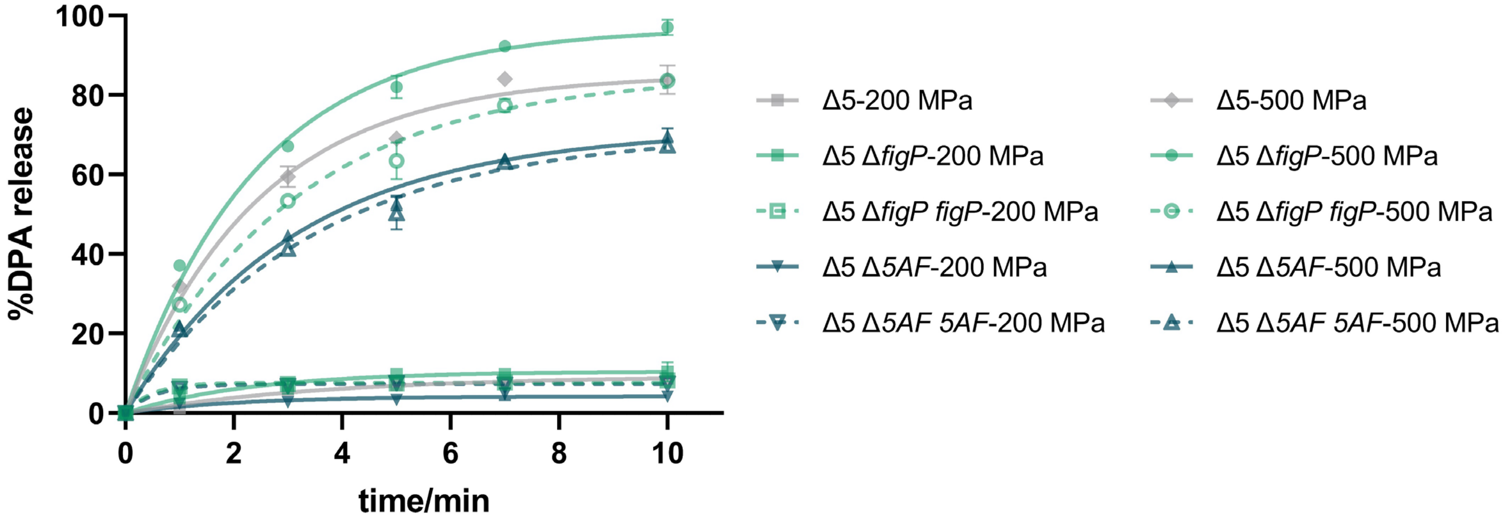

%DPA release
100
80
60
40
20
0
0
2
4
6
8
10
time/min
Δ5-200 MPa
Δ5 Δ*figP*-200 MPa
Δ5 Δ*figP figP*-200 MPa
Δ5 Δ*5AF*-200 MPa
Δ5 Δ*5AF 5AF*-200 MPa
Δ5-500 MPa
Δ5 Δ*figP*-500 MPa
Δ5 Δ*figP figP*-500 MPa
Δ5 Δ*5AF*-500 MPa
Δ5 Δ*5AF 5AF*-500 MPa

Figure S6

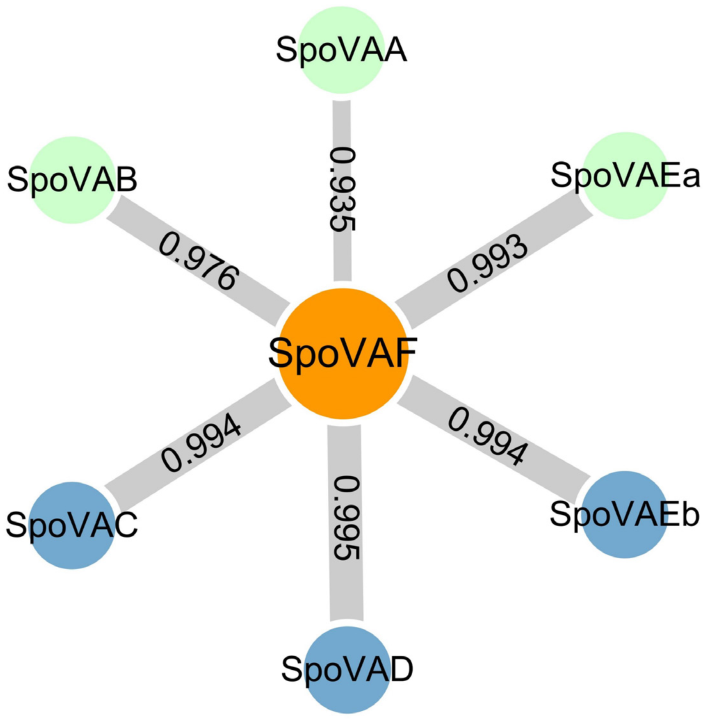
SpoVAA
SpoVAB
SpoVAEa
0.935
0.976
0.993
SpoVAF
0.994
0.994
0.995
SpoVAC
SpoVAEb
SpoVAD

Table S1. Strains used in this study.

Strain	Genotype	Source
PY79	Wild type	Lab stock
PS3551	*B. cereus* T (wild type)	a gift from Prof. Peter Setlow (UConn Health), originally from HO Halvorson
bLA201	*ΔgerA::lox72 ΔgerBB::lox72 ΔgerKB::lox72 ΔyfkT::lox72 ΔyndE::lox72*	a gift from Prof. David Rudner (Harvard Medical School) [1]
TYZ12	*ΔgerA::lox72 ΔgerBB::lox72 ΔgerKB::lox72 ΔyfkT::lox72 ΔyndE::lox72, amyE::gerA-cm*	This work
TYZ17	*ΔgerA::lox72 ΔgerBB::lox72 ΔgerKB::lox72 ΔyfkT::lox72 ΔyndE::lox72, amyE::gerA (B. cereus)-cm*	This work
YZ03	*ΔfigP::kan*	This work
YZ05	*ΔfigP::kan, amyE::*P_{figP}*-figP-cm*	This work
YZR33	*ΔgerA::lox72 ΔgerBB::lox72 ΔgerKB::lox72 ΔyfkT::lox72 ΔyndE::lox72 ΔfigP::kan*	This work
YZR34	*ΔgerA::lox72 ΔgerBB::lox72 ΔgerKB::lox72 ΔyfkT::lox72 ΔyndE::lox72 ΔfigP::kan, amyE::gerA-cm*	This work
YZR37	*ΔgerA::lox72 ΔgerBB::lox72 ΔgerKB::lox72 ΔyfkT::lox72 ΔyndE::lox72 ΔfigP::kan, amyE::gerA (B. cereus)-cm*	This work
YZR39	*ΔgerA::lox72 ΔgerBB::lox72 ΔgerKB::lox72 ΔyfkT::lox72 ΔyndE::lox72 ΔspoVAF::kan*	This work
KP09	*ΔspoVAF::kan*	This work
KP33	*ΔgerA::lox72 ΔgerBB::lox72 ΔgerKB::lox72 ΔyfkT::lox72 ΔyndE::lox72 ΔfigP::kan, amyE::*P_{figP}*-figP-cm*	This work
KP39	*ΔgerA::lox72 ΔgerBB::lox72 ΔgerKB::lox72 ΔyfkT::lox72 ΔyndE::lox72 ΔspoVAF::kan, amyE::*P_{spoVAA}*-spoVAF-cm*	This work

Detailed description of strain construction

TYZ12 (*ΔgerA::lox72 ΔgerBB::lox72 ΔgerKB::lox72 ΔyfkT::lox72 ΔyndE::lox72, amyE::gerA-cm)* was generated by transforming BLA201 with pTYZ12.

TYZ17 (*ΔgerA::lox72 ΔgerBB::lox72 ΔgerKB::lox72 ΔyfkT::lox72 ΔyndE::lox72, amyE::gerA from B.cereus-cm*) was generated by transforming BLA201 with pTYZ17

YZ03 (*ΔfigP::kan)*: PY79 was transformed with genomic DNA of strain BKK24480.

YZ05 (*ΔfigP::kan, amyE::*P_{figP}*-figP-cm*): YZ03 was transformed with pYZ05.

YZR33 (*ΔgerA::lox72 ΔgerBB::lox72 ΔgerKB::lox72 ΔyfkT::lox72 ΔyndE::lox72 ΔfigP::kan*): BLA201 was transformed with genomic DNA of strain BKK24480.

YZR34 (*ΔgerA ΔgerBB ΔgerKB ΔyfkT ΔyndE ΔfigP::kan, amyE::gerA-cm*): TYZ12 was transformed with genomic DNA of strain BKK24480.

YZR37 (*ΔgerA ΔgerBB ΔgerKB ΔyfkT ΔyndE ΔfigP::kan, amyE::gerA from B. cereus-cm*): TYZ17 was transformed with genomic DNA of strain BKK24480.

YZR39 (*ΔgerA ΔgerBB ΔgerKB ΔyfkT ΔyndE ΔspoVAF::kan*): BLA201 was transformed with genomic DNA of strain BKK23390.

KP09 (*ΔspoVAF::kan*): PY79 was transformed with genomic DNA of strain BKK23390.

KP33 (*ΔgerA::lox72 ΔgerBB::lox72 ΔgerKB::lox72 ΔyfkT::lox72 ΔyndE::lox72 ΔfigP::kan, amyE::*P_{figP}*-figP-cm*): YZR33 was transformed with pYZ05.

KP39 (*ΔgerA::lox72 ΔgerBB::lox72 ΔgerKB::lox72 ΔyfkT::lox72 ΔyndE::lox72 ΔspoVAF::kan, amyE::*P_{spoVAA}*-spoVAF-cm*): YZR39 was transformed with pKP39.

Table S2. Plasmids used in this study.

Plasmid	Description	Source
pDG364	*amyE::cm*	Guerout-Fleury et al., (1996) [2]
pTYZ12	*amyE::gerA-amp*	This work
pTYZ17	*amyE::gerA from B. cereus-amp*	This work
pYZ05	*amyE::*P_{figP}*-figP-cm*	This work
pKP39	*amyE::*P_{spoVAA}*-spoVAF-cm*	This work

Detailed description of plasmid construction

pTYZ12 (*amyE::gerA-amp*) containing the *gerA* gene (promoter and ORF) with flanking *amyE* sequences and a *amp* gene, was constructed by amplifying the *gerA* gene by PCR using primers oTYZ11-oTYZ12. The PCR-amplified DNA was cloned into the BamHI site of pDG364 (*amyE::cm*) using Gibson Assembly Kit (NEB, USA).

pTYZ17 (*amyE::gerA from B.cereus-amp)* containing the *gerA* gene (promoter and ORF) from *B. cereus* T (PS3551) with flanking *amyE* sequences and a *amp* gene, was constructed by amplifying the *gerA from B.cereus* gene by PCR using primers oTYZ24-oTYZ25. The PCR-amplified DNA was cloned into the BamHI site of pDG364 (*amyE::cm*) using Gibson Assembly Kit (NEB, USA).

pYZ05 (*amyE::*P_{figP}*-figP-cm*): containing the *figP* gene (promoter and ORF) with flanking *amyE* sequences and a *cm* gene, was constructed by amplifying the *yqhR* gene by PCR using primers oYZ051-oYZ052. The PCR-amplified DNA was cloned into the BamHI site of pDG364 (*amyE::cm*) using Gibson Assembly Kit (NEB, USA).

pKP39 (*amyE::*P_{spoVAA}*-spoVAF-cm*): containing the *spoVAF* gene and *spoVAA* promoter with flanking *amyE* sequences and a *cm* gene, was constructed by amplifying the *spoVAF* gene by PCR using primers oKP391 and oKP392, and *spoVAA* promoter gene by oKP393 and oKP394. The PCR-amplified DNA was cloned into the BamHI site of pDG364 (*amyE::cm*) using Gibson Assembly Kit (NEB, USA).

Table S3. Primers used in this study.

Primer	Sequence
oTYZ11	CCAACTGGTAATGGTAGCGACCGGCGCTCAGGCCATACTGTGCTTGAGAAA
oTYZ12	GTCAAACATGAGAATTCGATAAGCTTCTAGTTACGTTTCCGCCATTCTTTGC
oTYZ24	CCAACTGGTAATGGTAGCGACCGGCGCTCAGTTTGTATCCTCCGCTTTCATAAAA
oTYZ25	GTCAAACATGAGAATTCGATAAGCTTCTAGCTATTTGTTTGCGCCTTTCGT
oYZ051	CCAACTGGTAATGGTAGCGACCGGCGCTCAGCAGGAAACAGCGCTAAACCTCTA
oYZ052	GTCAAACATGAGAATTCGATAAGCTTCTAGCTATTCTCTATGCATTCCCAGCGC
oKP391	ATGCCGGACCACAAGGAAG
oKP392	GTCAAACATGAGAATTCGATAAGCTTCTAGTTATGAATTGGTAGGCTGCCTTAAGC
oKP393	CCAACTGGTAATGGTAGCGACCGGCGCTCAGTCTGCAGTGCAGGCTAGC
oKP394	CTTCCTTGTGGTCCGGCATCATTGATCACCATCTTTCGGTGG

Reference

[1] Artzi, Alon, Brock, Green, Tam, Ramírez-Guadiana, Marks, Kruse, Rudner. Dormant spores sense amino acids through the B subunits of their germination receptors. Nature Communications. 2021;12(1):6842. doi: 10.1038/s41467-021-27235-2.

[2] Guérout-Fleury, Frandsen, Stragier. Plasmids for ectopic integration in *Bacillus subtilis*. Gene. 1996;180(1):57-61. doi: 10.1016/S0378-1119(96)00404-0.
